# Protein diets with the role of immune and gut microbial regulation alleviate DSS‐induced chronic ulcerative colitis

**DOI:** 10.1002/fsn3.1914

**Published:** 2021-01-22

**Authors:** Shenyan Ma, Cheng Qian, Nan Li, Zhifeng Fang, Jianxin Zhao, Hao Zhang, Wei Chen, Zhenmin Liu, Wenwei Lu

**Affiliations:** ^1^ State Key Laboratory of Food Science and Technology Jiangnan University Wuxi China; ^2^ School of Food Science and Technology Jiangnan University Wuxi China; ^3^ State Key Laboratory of Dairy Biotechnology Shanghai Engineering Research Center of Dairy Biotechnology Dairy Research Institute Bright Dairy & Food Co., Ltd. Shanghai China; ^4^ National Engineering Research Center for Functional Food Jiangnan University Wuxi China; ^5^ Wuxi Translational Medicine Research Center and Jiangsu Translational Medicine Research Institute Wuxi Branch Wuxi China; ^6^ Beijing Innovation Centre of Food Nutrition and Human Health Beijing Technology and Business University (BTBU) Beijing China

**Keywords:** chronic ulcerative colitis, gut microbiota, immune regulation, protein diets

## Abstract

The association between diet and inflammatory bowel disease (IBD) has been confirmed. However, the role of protein consumption in IBD remains controversial. This research aimed to explore the effects of milk‐based protein (MBP), potato protein (PP), and mixed protein (MP) on the recovery of mice with dextran sulfate sodium (DSS)‐induced ulcerative colitis (UC). MP alleviated UC symptoms through reducing colon shortening and tissue damage, decreasing neutrophil infiltration, maintaining the mucous layer integrity, and suppressing the expression of TNF‐α, IL‐17A, IL‐6, and IL‐1β. MBP and PP decreased the colon shortening and IL‐1β levels but PP increased the MUC2 expression. Additionally, the gut microbial structure and composition were altered after different proteins supplement. Compared to DSS‐treated mice, MP‐treated mice showed that increased abundances in *Coprococcus* and *Bifidobacterium* and decreased abundances in *Sutterella*, *Lactobacillus*, and *Akkermansia*. MBP increased the proportion of *Bifidobacterium* and reduced *Sutterella*, but PP increased *Ruminococcus* and *Bifidobacterium* and decreased *Adlercreutzia*. Correspondence analysis of gut microbial composition to determine the effects of protein diets on immune response and pathological characteristics also verified the interaction between gut microbiota and alleviation of colitis. These results provide a theoretical basis for the selection of raw materials for clinical enteral nutrition preparations and potential use for potato protein wastes.

## Graphical abstract

Protein diets alleviate the pathological features of chronic ulcerative colitis. Protein diets regulated the immune responses in mice with chronic ulcerative colitis. Protein diets altered the gut microbial structure and composition in mice with chronic ulcerative colitis.

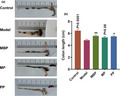

## 1 INTRODUCTION

Inflammatory bowel disease (IBD) is chronic and recurrent, which includes ulcerative colitis (UC) and Crohn's disease (CD) (Baumgart & Carding, 2007). The prevalence of IBD has increased rapidly in modern times because of unreasonable dietary structure, increased work pressure, and environmental degradation (Kaplan & Ng, 2017). The pathogenesis of IBD remains somewhat unclear, although various contributing factors have been identified, including genetics, infection, intestinal mucosal barrier function, environment, and immunity (Ng et al., 2018). Although currently available treatments such as immunosuppressants, aminosalicylic drugs, cortisol drugs, and antibiotics have yielded certain benefits, they are associated with serious side effects and are not suitable for long‐term use (Khanna et al., 2013). In contrast, reasonable dietary regulation may be a feasible approach to relieve UC.

The diet plays an important role in maintaining the gut microenvironment through modulating effects on the microbial composition and function, gut barrier integrity, and host immunity (Levine et al., 2018). Numerous epidemiological studies have demonstrated the correlation between intake of specific dietary ingredients or dietary patterns and the risk of developing IBD. Several studies have indicated a causal link between the Western dietary pattern and a high incidence of colorectal cancer, whereas other studies have proven a beneficial effect of the Mediterranean diet on IBD (Donovan et al., 2017). Among the available animal‐derived protein, the high intake of meat or fish rather than eggs or dairy products was linked with a high incidence of IBD (Jantchou et al., 2010). Protein‐energy malnutrition accompanied by lack of protein is common in adult patients with IBD, which mainly presents as weight loss. In children, however, protein‐energy malnutrition can even stunt growth (Hengstermann et al., 2008). Although the nutritional values of food proteins vary because of differences in amino acid compositions, the World Health Organization/Food and Agriculture Organization of the United Nations/United Nations University (WHO/FAO/UNU) has indicated that protein safety requirements should be based on the consumption of high‐quality protein and sufficient energy supply (Protein and amino acid requirements in human nutrition, 2007).

A high‐quality protein contains a complete amino acid profile with sufficient quantities and appropriate proportions of amino acids, as well as an amino acid pattern similar to that of human proteins. This latter characteristic ensures that the protein will be easily absorbed by the human body. However, the role of protein consumption in IBD remains controversial. Studies have demonstrated a causal link between the high intake of animal proteins (except dairy proteins) and IBD, as the heme and amino acids in meat are not absorbed by the small intestine and induce the production of harmful substances in the colon (Windey et al., 2012). In Japan, researchers have reported that an increased intake of dairy products and meat is associated with an increasing trend in the prevalence of UC (Ng, 2014). One promising explanation for this phenomenon suggests that an allergy to cow's milk proteins during infancy is responsible for the development of CD and UC later in childhood (Virta et al., 2013). Gorissen et al. have compared the amino acid composition of proteins for daily intake and observed that among plant sources, potato protein is the best in compliance with the WHO/FAO/UNU‐recommended requirements for all essential amino acids. Interestingly, the essential amino acid content of potato protein even exceeded that of casein (Gorissen et al., 2018). Currently, potato protein is often discarded as waste by the potato starch‐processing industry, which represents a great waste of a useful resource (Członka et al., 2018). Therefore, this study aimed to compare the effects of milk‐derived protein, plant‐derived protein, or their mixture on the clinical symptoms in mice with DSS‐induced UC.

## 2 MATERIALS AND METHODS

### 2.1 Animal experimental design

The animal experiment protocol was approved by the Animal Ethics Committee of Jiangnan University (JN.No20180615c1101030). C57BL/6J mice (male, age: 8 weeks, body weight: 18–20 g, Shanghai SLAC Laboratory Animal Co., Ltd.) were housed in a climate‐controlled room (specific pathogen‐free, 20‐26°C, 40%‐70% humidity, and a 12‐hr light–dark cycle) in the Animal Experiment Center of Jiangnan University.

After a 1‐week adaptation, 50 mice were randomly divided into five equal groups (*n* = 10): control group, model group, and three experimental groups. To induce chronic colitis, all groups except the control group (control mice) were administered 2%–3% DSS (wt/vol, 36–50 kDa, MP Biomedicals) in their drinking water for three cycles (7 days DSS induction, 14 days water per cycle) (Wirtz et al., 2017). Two weeks before the end of the experiment, the three experimental groups were fed a diet containing milk‐based protein (MBP, whey protein: casein = 1:1), potato protein (PP), or mixed protein (MP, whey protein:casein: potato protein = 1:1:1, Shanghai Chuangshi Co., Ltd.) ad libitum for 2 weeks. These diets are described in Table 1, and all protein‐supplemented diets provided the same amount of energy. The control and model groups were fed standard commercial chow (Shoobree, Jiangsu Xietong Pharmaceutical Bio‐engineering Co., Ltd., 21.8% crude protein and 5% crude fat). At the end of the trial, the mice were sacrificed, fecal contents were collected from the colon of the mice, and the colons were also collected. The length of each colon was measured, and a 5‐mm sample was cut and fixed in a 4% paraformaldehyde solution. The remaining colon tissue was frozen at −80°C until analysis.

**TABLE 1:** The major nutritional compositions of different feeds

Nutrition	Content/kg		
	MBP	MP	PP
Protein			
Whey protein (g)	92.19	61.46	‐
Casein (g)	92.19	61.46	‐
Potato protein (g)	‐	61.46	184.38
Fat			
Soybean oil (g)	62.29	62.29	62.29
MCT (g)	62.29	62.29	62.29
Carbohydrate			
Maltodextrin (g)	609.06	609.06	609.06
Inulin (g)	40	40	40
Multi‐minerals (g)	50	50	50
Multi‐vitamins (g)	5	5	5

### 2.2 Changes in colon length

After the sacrifice, the colon was collected and the entire length was measured.

### 2.3 Histology staining

Ultrathin slices (5 μm) were obtained by cutting the paraffin‐embedded colon samples (Yang et al., 2018). The slices were then stained with hematoxylin and eosin (H&E, Yulu Laboratory Equipment Co., Ltd.) to evaluate the degree of inflammation as indicated by the presence of mucosal ulceration, dysplasia, and immune cell infiltration (Dieleman et al., 1998). Stained sections were observed using a light microscope (BA210, Motic China Group Co., Ltd.).

### 2.4 Myeloperoxidase activity measurement

Myeloperoxidase (MPO) activity in colonic tissue was assayed using a myeloperoxidase assay kit (Nanjing Jiancheng Co., Ltd.) according to the manufacturer's instructions.

### 2.5 Immune indicators expression measurement

Immune indicators expression in colonic tissue homogenates was evaluated using an enzyme‐linked immunosorbent assay (ELISA) kit (R&D Systems). Briefly, dissected tissues were homogenized in RIPA buffer containing protease inhibitors (Boston Bioproducts). The protein concentrations in the homogenates were determined using the bicinchoninic acid assay. The concentrations of interleukin (IL)‐1β, IL‐6, IL‐17A, and tumor necrosis factor (TNF)‐α were analyzed using according to the manufacturer's instructions.

### 2.6 Cell apoptosis

Ultrathin slice (5 µm) was stained with Hoechst 33,258 according to the manufacturer's instructions (Shanghai Beyotime Biotechnology Co., Ltd.) and assessed using a light microscope to determine the apoptosis of intestinal epithelial cells (IECs).

### 2.7 Alcian blue staining

Ultrathin slices (5 μm) were stained with alcian blue according to the manufacturer's instructions (Nanjing SenBeiJia Biological Technology Co., Ltd) (Yan et al., 2019). Stained sections were observed using a light microscope.

### 2.8 MUC2 expression analysis

MUC2 expression in the colonic tissue homogenates was analyzed using ELISA according to the manufacturer's instructions (Nanjing SenBeiJia Biological Technology Co., Ltd).

### 2.9 16S rRNA amplification‐based sequencing of the gut microbiota

Bacterial DNA was extracted from fecal samples by a FastDNA Spin Kit for Feces (MP Biomedicals) according to the manufacturer's instructions. The V3‐V4 region was amplified and sequenced using the Illumina MiSeq platform (Illumina) (Tian et al., 2019). Briefly, PCR products were excised from a 2.0% agarose gel (Sangon Biotech, Sangon Biotech [Shanghai] Co., Ltd.) and purified using TIANgel mini purification kit (BIOMIGA, Hangzhou Biomiga technology Co., Ltd.). Libraries were prepared using TruSeq DNA LT sample preparation kits (Illumina) and sequenced for 500 + 7 cycles on the Miseq platform (Illumina) using the Miseq reagent kit (Illumina). 16S rRNA sequence data were measured using the QIIME pipeline (open‐source, http://qiime.org/). Sequences with similarity >97% were clustered into operational taxonomic units (OTU), and representative sequences of each cluster were used to classify bacterial taxa.

### 2.10 Statistical analysis

Statistical analyses were performed using GraphPad Prism 8.0 (GraphPad Inc.). All data were presented as the mean ± *SEM* and were analyzed using one‐way analysis of variance (ANOVA) followed the post hoc Fisher's least significant difference test (*p < .05, **p < .01, and ***p < .001 versus. the model group). A *p* value of ≤.05 was considered indicative of statistical significance. The Bray–Curtis index was used to perform nonmetric multi‐dimensional scaling (NMDS). A linear discriminant analysis (LDA) effect size (LEfSe) was performed to identify microbial biomarkers. To examine the relationship between parameters associated with colitis and gut microbial alteration, a principal component analysis (PCA) was carried out using XLSTAT (Addinsoft) (Kwon et al., 2018).

## 3 RESULTS

### 3.1 Protein diets alleviated colitis symptoms

**FIGURE 1:**
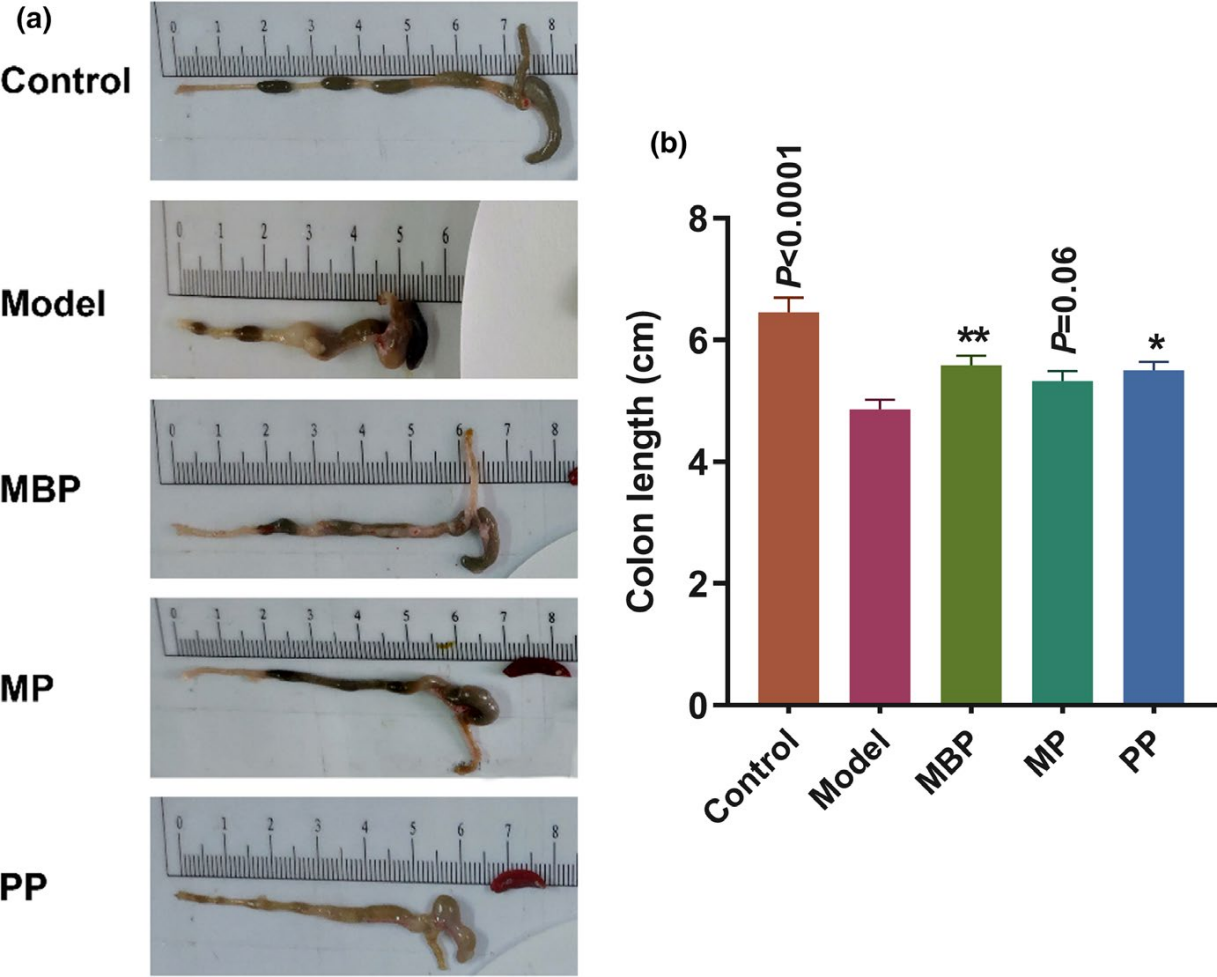
Effects of protein diets on colitis symptoms. (a) Representative colons from mice in different groups; (b) Change in colon length

Colitis leads to a significant shortening of the colon in mice. Therefore, colon length shortening is used as a macroscopic indicator of colon injury. The significantly decreased colon length of mice was induced by DSS in the model group compared to the control group (Figure 1a,b). Compared to the model group, the mice in all three protein‐based dietary intervention groups showed reduced symptoms of colon length shortening, although there was no statistical significance in the MP group (Figure 1a,b). MBP and PP intervention significantly suppressed the inflammation of the colon induced by DSS and thus reduced the shortening of the colon. The results showed milk‐derived protein, plant‐derived protein, and their mixture had the potential to alleviate colitis symptoms.

### 3.2 Protein diets suppressed the inflammation of colitis

**FIGURE 2:**
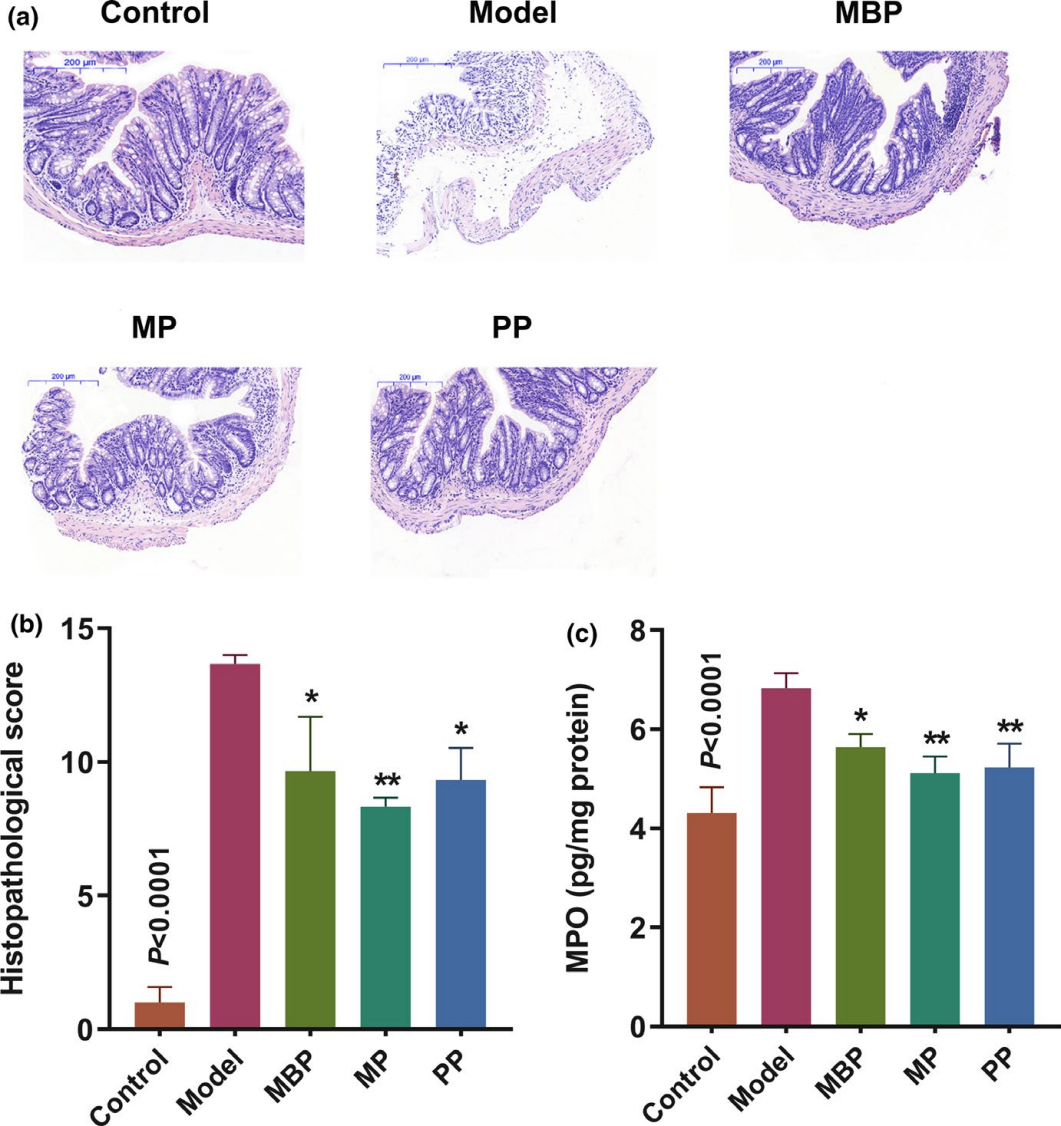
Effects of protein diets on intestinal inflammation. (a) Representative histological images of H&E‐stained tissues. Scale bars, 200 μm; (b) colonic histological scores; (c) myeloperoxidase activity

To evaluate the effects of dietary proteins on histopathology of colonic tissues, H&E staining was used to analyze changes in pathological features of the colon. DSS‐treated mice exhibited severe inflammation, including crypt distortion, goblet cell losses, severe epithelial damage, and inflammatory cell infiltration of the mucosa (Figure 2a). However, mice treated with dietary proteins exhibited considerably reduced symptoms, with only mild tissue damage and inflammation. Additionally, the protein‐treated group had a significantly reduced histological injury score versus the model group (*p* < .05; Figure 2b).

Myeloperoxidase is an enzyme expressed in granulocytes (e.g., neutrophils). As neutrophil enrichment is usually associated with inflammation, the level of MPO activity in the colon is positively correlated with the severity of granulocyte infiltration (Chassaing et al., 2014). As shown in Figure 2c, DSS‐treated mice exhibited a notably increase in the MPO activity compared to the mice in the control group. However, all three dietary proteins significantly decreased MPO expression (*p* < .05). These results showed that milk‐derived protein, plant‐derived protein, and their mixture significantly suppressed aggravation and deterioration of the colon and contributed to the alleviation of inflammatory infiltration.

### 3.3 Protein diets regulated the immune responses

**FIGURE 3:**
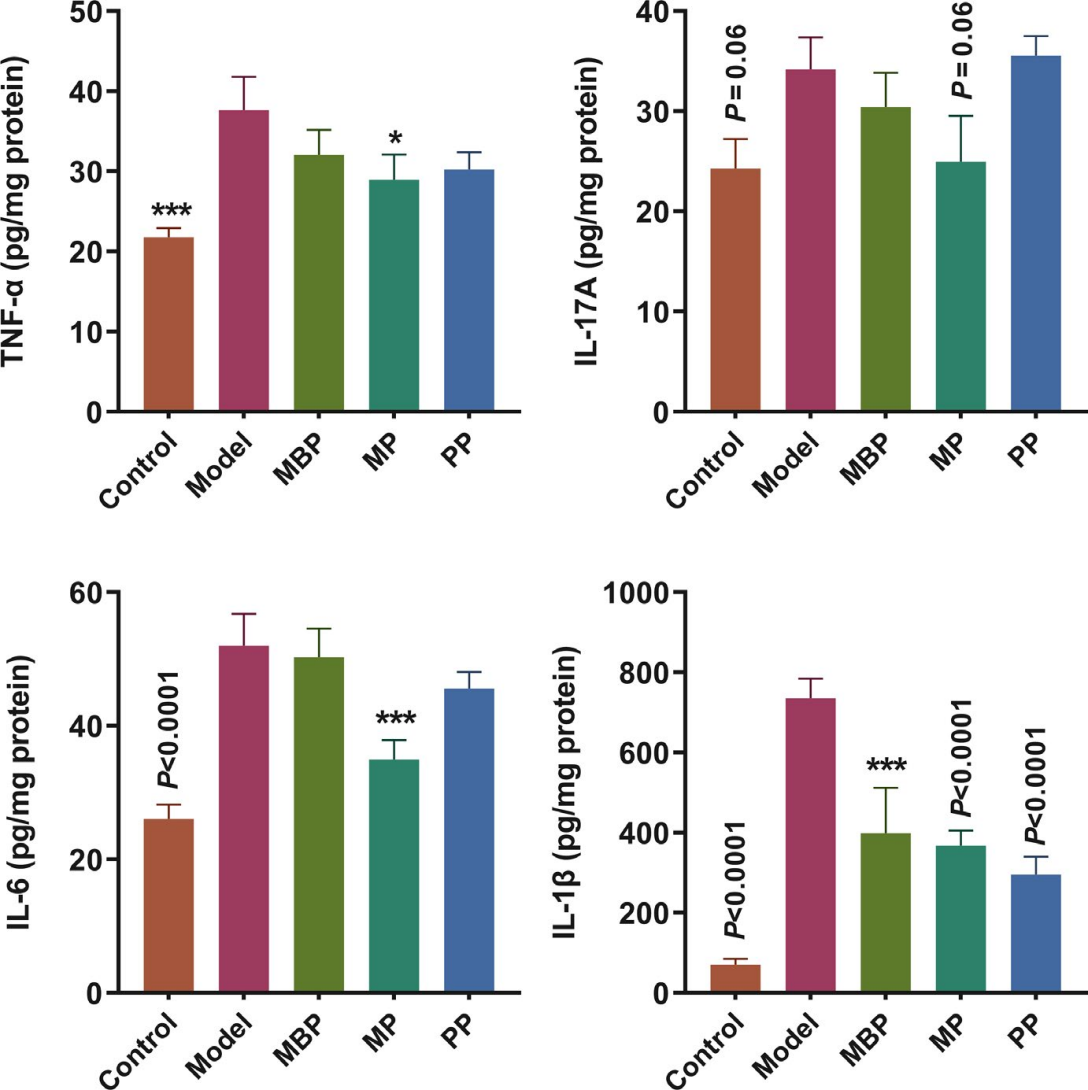
Effects of protein diets on the levels of inflammatory cytokines (IL‐17A, IL‐6, TNF‐α, and IL‐1β)

The increased secretion and infiltration of pro‐inflammatory cytokines contribute to the development of DSS‐induced colitis. To explore changes in the immune responses after protein diets intervention, cytokine expression in colon tissue was measured. As shown in Figure 3, the colonic tissues of the DSS‐treated mice exhibited significantly increased TNF‐α, IL‐6, IL‐17A, and IL‐1β compared with those in the control mice. MP also decreased all four pro‐inflammatory cytokines expression. MBP and PP significantly reduced the IL‐1β levels but could not decrease TNF‐α, IL‐17A, and IL‐6. Therefore, MP was more efficient to suppress the pro‐inflammatory responses than MBP and PP. The results showed that protein diets interventions contributed to the regulation of the immune responses in the colon and the mixed protein had the more potential to maintain the integrity of the intestinal barrier than MBP and PP.

### 3.4 Protein diets improved the intestinal epithelial barrier

**FIGURE 4:**
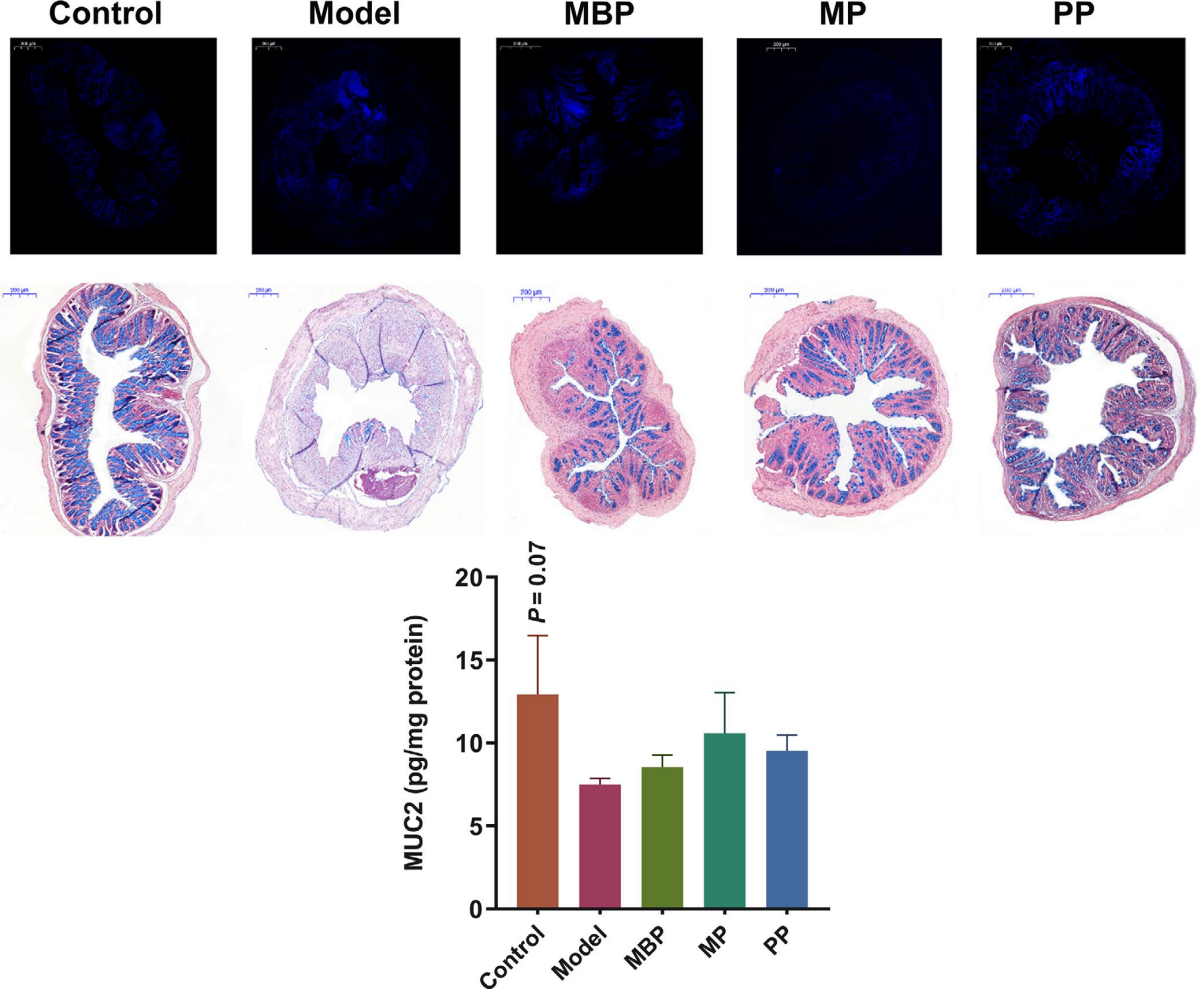
Effects of protein diets on the intestinal epithelial barrier. Cell apoptosis (top); Alcian blue staining. Scale bars, 200 μm (middle); MUC2 expression (bottom)

A morphological analysis of Hoechst‐stained tissues revealed that control cells were round and light blue, whereas apoptotic cells were bright blue because of an increase in cell density or the fluorescent‐labeling of fragmented nuclei. As shown in Figure 4 (top), the cells in colonic tissues from the control mice were neatly arranged and exhibited a light blue color, whereas those of the DSS‐treated mice showed more bright blue areas with severe apoptosis. The colonic tissues of MBP‐ and PP‐treated mice mostly exhibited a light blue color and slight apoptosis, but similar to the control group, MP treatment showed a light blue color and neatly arranged cells.

The intestine is covered with a layer of mucus that functions as a protective physical barrier. alcian blue staining (Vector) were used to observe the distribution of mucin in the colon. In the model group, the mucous layer had disappeared, and severe goblet cell destruction was observed (Figure 4, middle). In contrast, all dietary proteins provided strong protection for the goblet cells and mucosa in the treated mice. Several mucins comprise the gel‐forming glycoprotein component of this barrier. It was reported that MUC2 mainly contributed to the colonic mucus layer among these mucins (Kumar et al., 2017). As shown in Figure 4 (bottom), the protein‐treated mice, particularly those treated with MP and PP, exhibited a trend toward enhanced MUC2 expression. Collectively, these results showed that protein diets intervention contributed to a decrease in intestinal permeability and thus improved the functions of the intestinal barrier.

### 3.5 Protein diets modulated gut microbial diversity and composition

**FIGURE 5:**
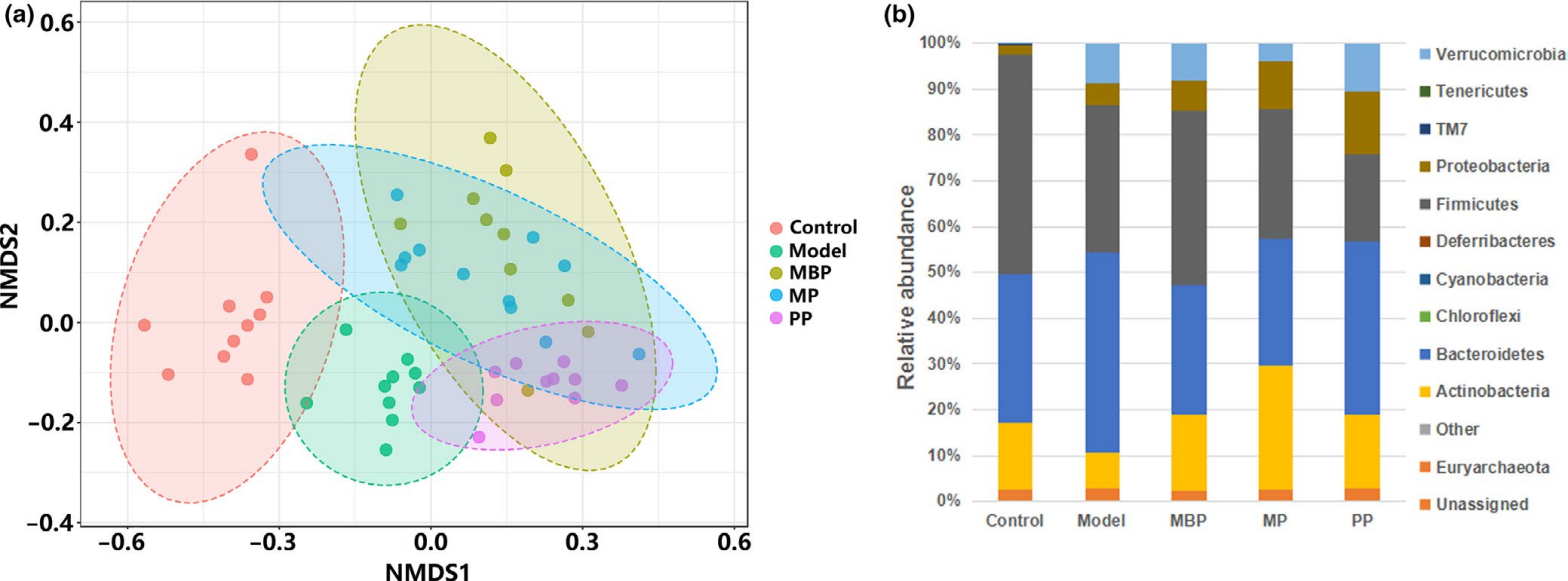
Effects of protein diets on the gut microbial structure and composition. (a) Beta diversity; (b) changes in the phylum‐level microbial composition

To investigate the effects of protein diets on the gut microbial structure and composition, 16S rRNA amplification sequencing was performed. The gut microbial β diversity was analyzed using NMDS based on the Bray–Curtis index. The results showed that the control and model groups were separated and the differences between both two groups. The protein diets treatments remarkably affected the gut microbial structure and composition (Figure 5a). The dominant phyla in the control mice were *Verrucomicrobia* (0.047%), *Bacteroidetes* (32.53%), *Firmicutes* (47.95%), *Actinobacteria* (14.50%), and *Proteobacteria* (2.03%) (Figure 5b). DSS treatment significantly altered gut microbial composition, as indicated by increases in the relative abundances of *Bacteroidetes*, *Verrucomicrobia*, and *Proteobacteria* to 43.85%, 8.58%, and 4.93%, respectively, and decreases in the relative abundances of *Firmicutes* and *Actinobacteria* to 32.04% and 7.66%, respectively, in the model group. Accordingly, the *Firmicutes*/*Bacteroidetes* (F/B) ratio decreased from 147.38% to 73.08% after DSS treatment. MBP‐ and MP‐treated mice showed significantly increased F/B ratios of approximately 134.75% and 101.91%, respectively, compared to the DSS‐treated mice.

**FIGURE 6:**
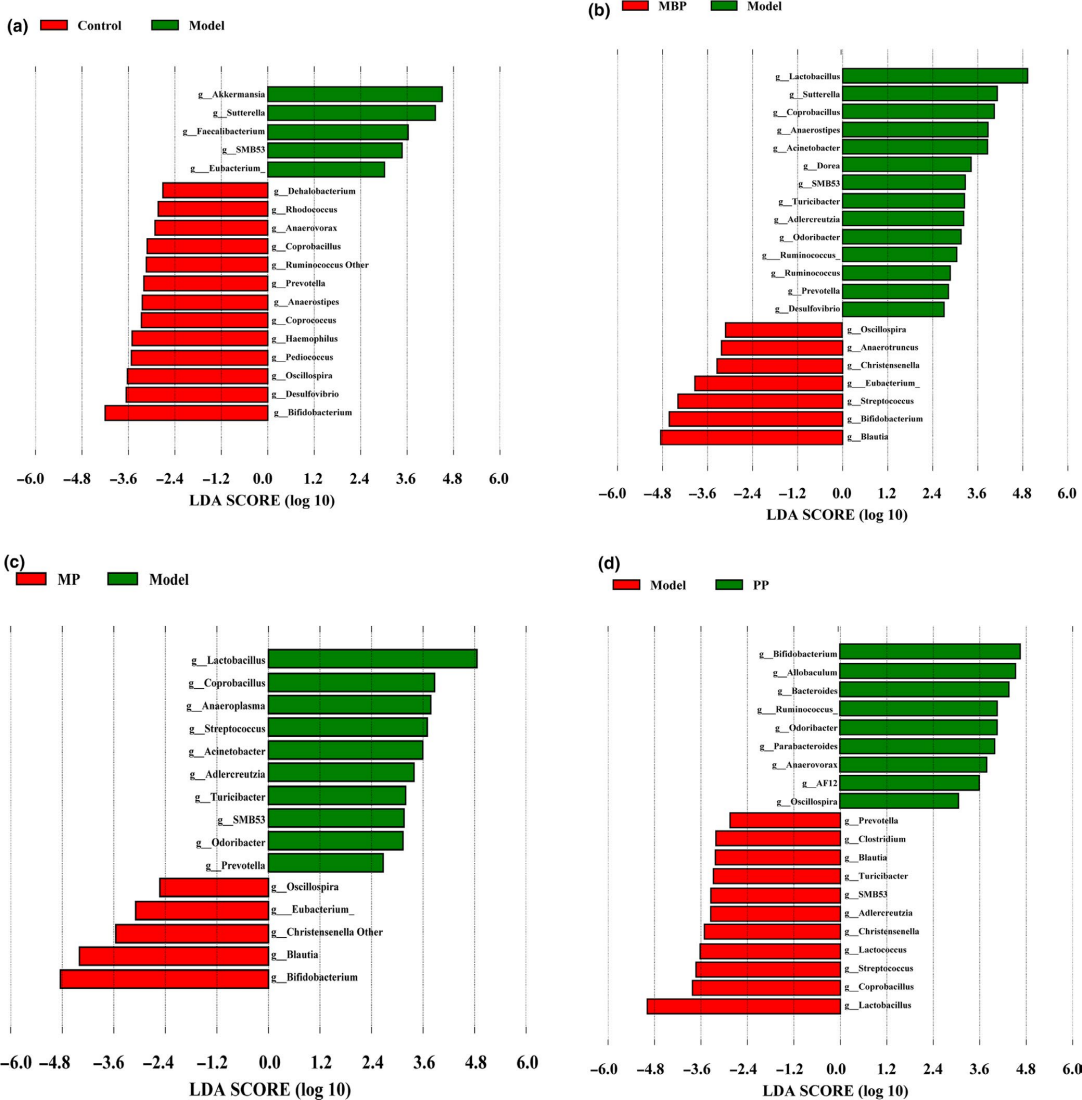
Linear effect size comparison between the model group and other groups (*p* < .05, LDA score (log10) >2.0)

**FIGURE 7:**
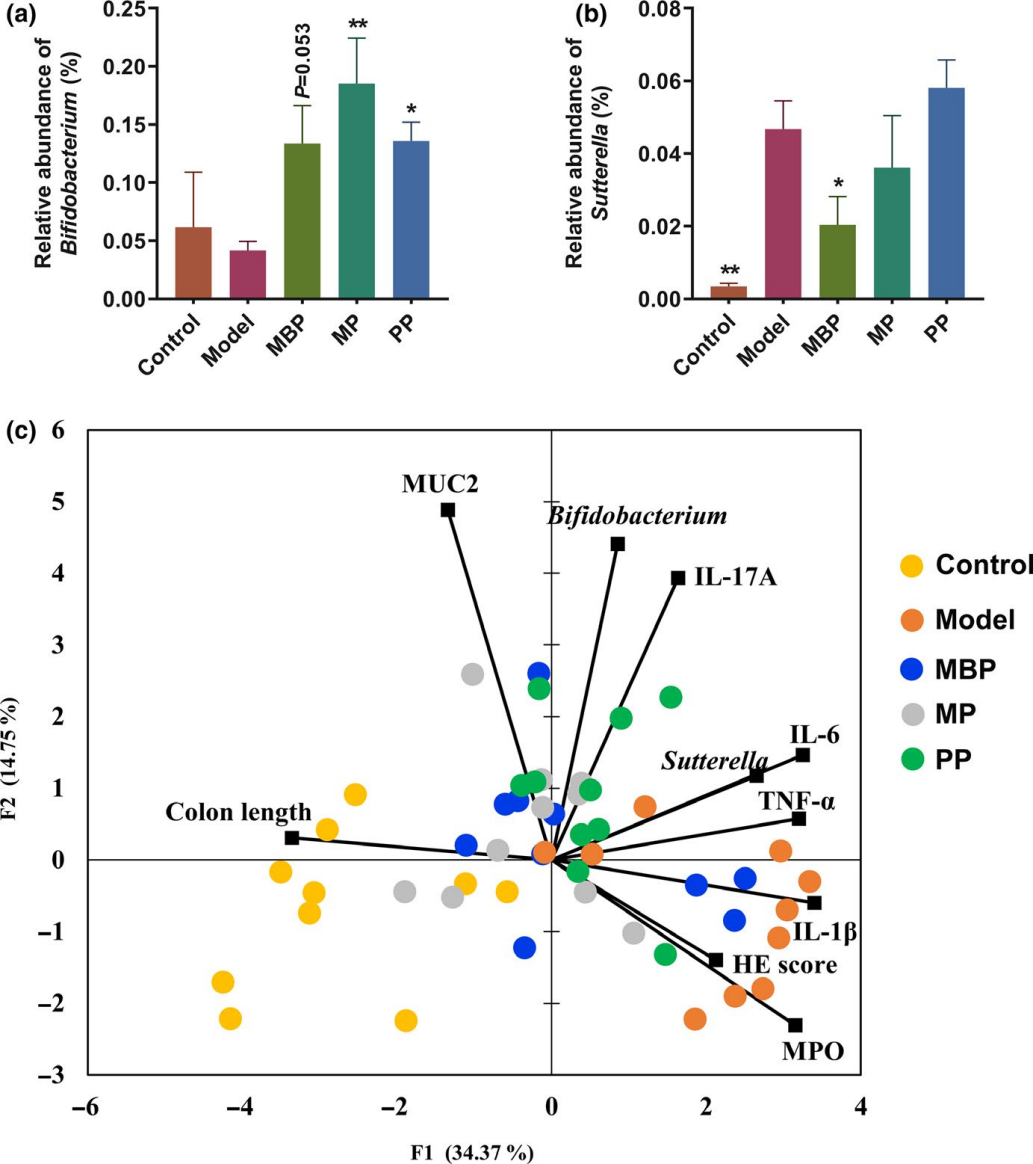
Relationships between the investigated variables. (a) Changes in the proportions of *Bifidobacterium* and *Sutterella*; (b) correspondence analysis of fecal microbiome composition and investigated variables

To identify the biomarkers after protein diets treatments in all groups, LEfSe was performed. The cladogram indicated differences in taxa between all groups (*p* < .05, LDA score (log10) >2, Wilcoxon rank‐sum test, Figure 6). Notably, differences in the relative abundances of the genera *Prevotella*, *Oscillospira*, *Faccalibacterium*, *Coprobacillus*, *Anaerostipes*, *Pediococcus*, *Anaerovorax*, *Desulfovibrio*, *Dehalobacterium*, *Haemophilus*, and *Rhodococcus* were observed between the DSS‐treated group and the control group (Figure 6a). However, no significant differences were observed between the DSS‐treated group and the other three experimental groups. No inter‐group differences were observed in the abundances of the genera *Acinetobacter*, *Dorea*, *Turicibacter*, *Clostridium*, and *Anaerotruncus*. Compared to DSS treatment alone, MBP treatment was associated with decreases in the relative abundances of *Sutterella*, *SMB53*, *Adlercreutzia*, and *Lactobacillus* and increases in those of *Coprococcus*, *Streptococcus*, *Bifidobacterium*, *Bacteroides*, *Lactococcus*, *Blautia*, *Christensenella*, and *Eubacterium* (Figure 5b). Dietary MP led to significant decreases in the relative abundances of *Lactobacillus*, *Sutterella*, and *Adlercreutzia* and increases in those of *Bifidobacterium*, *Parabacteroides*, and *Blautia* (Figure 6c). Dietary PP led to significant decreases in the relative abundances of the genera *Lactobacillus* and *Adlercreutzia* and increases in those of *AF12*, *Ruminococcus*, *Allobaculum*, *Odoribacter*, *Bifidobacterium*, *Parabacteroides*, and *Bacteroides* (Figure 6d). Additionally, all three protein diets significantly increased the proportion of *Bifidobacterium* but MBP and MP reduced the proportion of *Sutterella* compared to the model group (Figure 7a).

The results showed that protein diets treatments altered the gut microbial diversity and composition and thus demonstrated the interaction between changes in gut microbiota and alleviation of colitis.

### 3.6 Relationships between the investigated variables

Correspondence analysis of gut microbial composition to determine the effects of protein diets on immune response and pathological characteristics was performed (Figure 7b). Alteration in the abundance of genus *Sutterella* was positively correlated with pro‐inflammatory cytokines in colitis, whereas the change in *Bifidobacterium* was negatively correlated with histopathological score and MPO, especially induced by MP treatment. These results indicated that MP might restore immune balance in AD through modulation of the gut microbiota.

## 4 DISCUSSION

Malnutrition, especially protein‐energy malnutrition, is among the most important factors associated with a poor outcome in patients with IBD (Sandhu et al., 2016). This factor predominantly presents as weight loss and may also affect growth and development in affected children (Wędrychowicz et al., 2016). Therefore, this experimental study aimed at investigating the effects of different protein sources in the resolution of UC symptoms.

After administering dietary interventions based on different protein sources, the most significant increases in body weight were observed in MBP‐treated mice, followed by MP‐treated mice. In contrast, a downward trend in body weight was observed in the PP‐treated mice (unpublished). A previous report indicated that the digestibility of animal‐derived proteins (i.e., animal and dairy proteins) exceeded 90% and was normally higher than that of plant proteins (70%–90%). Lallés et al. observed that the replacement of skim milk powder with plant protein sources led to an apparent decrease in protein digestibility and impaired calf growth (Lallés, 1993). These results suggested that the better digestibility of MBP may be responsible for the superior body weight gain. The MBP‐treated mice exhibited amelioration of colon shortening symptoms and significant increases in the colon length versus the DSS‐treated mice (Figure 1). These results indicated that MBP and MP could relieve the symptoms of intestinal inflammation.

Inflammation comprises several components, including local and systemic manifestations. The early stages of this dynamic process are characterized by neutrophilic activity (Miossec & Kolls, 2012), the degree of which can be characterized by the level of MPO activity. In our study, the MPO activity decreased significantly in all dietary protein‐treated mice relative to the DSS‐treated mice (*p* < .05) (Figure 2). Additionally, there was no significant difference in MPO activity between the MP‐ and PP‐treated mice and control mice. These results indicated that both MP and PP could reduce neutrophil infiltration. Previously, Young et al. observed that a soy protein‐peptide could reduce the MPO activity in a model of DSS‐induced colitis, which was consistent with our findings (Young et al., 2012). Our analysis of H&E‐stained tissues and the associated tissue damage scores suggested that all dietary protein supplements significantly ameliorated the tissue damage associated with DSS‐induced UC. Moreover, MP‐ and PP‐treated mice exhibited lower tissue scores than MBP‐treated mice, although the differences were not significant. These observations indicated that both MP and PP exert beneficial effects concerning resolving tissue damage and reducing inflammatory cell infiltration.

Dextran sulfate sodium‐induced UC is associated with various clinical signs and the elevated expression of pro‐inflammatory cytokines, including IL‐6, IL‐17A, IL‐1β, and TNF‐α, which was consistent with the cytokine milieu observed in the mucosa of clinically active IBD patients (Eastaff‐Leung et al., 2010). In our study, PP treatment reduced the expression of IL‐1β but did not regulate the other three cytokines levels (Figure 3). In contrast, MP treatment suppressed the expression of most pro‐inflammatory cytokines. These results might be attributable to the low digestibility of plant protein, which is not completely absorbed by the small intestine. Protein fermentation in the colon can lead to the production of harmful substances such as hydrogen sulfide, which may promote colitis. Corpet et al. found that the addition of proteins that had been thermolyzed to reduce digestibility to the diets of rats increased protein fermentation in the colon (Corpet et al., 1995). In our study, the MP group received a lower dose of PP than the PP group; thus, a reduced amount of PP would have entered the colon. Therefore, the MP supplement provided certain, particularly in terms of the inhibition of the expression of pro‐inflammatory factors and consequent regulation of immunity.

Disruption of the intestinal epithelial barrier function is a common feature of UC and is associated with the abnormal apoptosis of IECs (Vetuschi et al., 2002). Our histological analysis revealed a decrease in apoptosis in the MP‐treated groups (Figure 4, top). However, MBP and PP treatments had no effects on apoptosis. TNF‐α secretion is associated with apoptosis, and inhibition of TNF‐α expression can reduce apoptosis (Chae et al., 2019). Therefore, MP treatment might reduce apoptosis by decreasing TNF‐α expression (Figure 3). Notably, all dietary protein supplementations reduced the loss of goblet cells (Figure 4, middle). Moreover, MP‐ and PP‐treated mice exhibited increased MUC2 expression relative to the DSS‐treated mice (Figure 4, bottom). A previous report indicated that whey protein promoted the synthesis of mucins and thus improved intestinal inflammation via a mechanism that may be attributable to threonine (Nichols & Bertolo, 2008). Threonine is excellent in potato protein (Liu et al., 2017) and Gorissen et al. determined the amino acid compositions of many proteins from different sources and observed a higher threonine content in PP than in MBP (Gorissen et al., 2018). In summary, these results indicated that the superior effects of MP and PP on MUC2 expression may be due to the action of threonine. Thus, MP treatment provided superior amelioration of the intestinal barrier damage.

Our results further demonstrated that different dietary proteins significantly modulated the microbiota composition in the DSS‐treated mice. Previous research identified a decrease in *Firmicutes* abundance and an increase in *Bacteroidetes* abundance in patients with IBD (Hu et al., 2019). We observed increases in the F/B ratios of MBP‐ and MP‐treated mice relative to that of DSS‐treated mice (Figure 5a). NMDS analysis revealed that different protein treatments caused different changes in the gut microbiota, and the structure in the MP‐treated group was more similar to that of the control group (Figure 5b).

Next, the complex relationships between IBD and certain gut microbiota were explored. The abundance of *Coprococcus*, a genus of butyrate‐producing bacteria, is decreased in patients with IBD (Kellermayer et al., 2015). The abundance of this genus was decreased in our DSS‐treated mice and increased in the MBP‐ and MP‐treated mice (Figure 6b,c), although the differences were not statistically significant. Moreover, we observed a significant decrease in the abundance of *Sutterella*, which is associated with digestive disorders (Mukhopadhya et al., 2011), in the MBP‐treated mice versus the DSS‐treated mice (Figure 6b). This observation is consistent with the lower digestibility of MP and PP (Figure 6c,d). The precise role and identity of the genus *Akkermansia* in chronic inflammatory conditions such as IBD remain to be established, as this genus exhibits both protective and deleterious actions (Anhê et al., 2016). In our study, A notable increase in the abundance of *Akkermansia* was observed in the DSS‐treated group versus the control group (Figure 6a), as well as a positive correlation between the abundance of this genus and IBD. Moreover, only the MP‐treated group showed a decreased abundance of *Akkermansia* relative to that in the DSS‐treated group, although the differences were not statistically significant. Furthermore, *Lactobacillus* and *Bifidobacterium* are well‐known to be beneficial for patients with IBD. Thus, our results were consistent with previous findings. Particularly, the abundance of *Bifidobacterium* increased significantly after MP and PP treatments.

Mixed protein was more effective in the recovery of the DSS‐treated mice than MBP and PP. This outcome resulted from better digestibility than MBP and PP. In the colon, undigested protein fermentation can exacerbate the symptoms of colitis and dysregulate the immune response. Therefore, the mixture of MP and PP (i.e., MP) reduced the intake of indigestible protein. Notably, PP contains a higher amount of threonine than MBP, which might help to ameliorate the intestinal barrier damage associated with UC. Finally, treatment with MP modulated the gut microbiota by increasing the abundances of *Coprococcus* and *Bifidobacterium* and decreasing those of *Sutterella* and *Akkermansia*. The correspondence analysis of gut microbial composition to determine the effects of protein diets on immune response and pathological characteristics also verified the interaction between gut microbiota and alleviation of colitis (Figure 7b). However, there are some limitations of this study, for example, the mechanism needs to be shown and the material basis (the effective substance‐derived gut microbiota) is still to be explored and measured. To understand the mechanisms of diets on UC, the relationship between diets and the colonic immune responses needs to be explored, such as the effects of diets on differentiation of colonic regulatory T‐cell and lipopolysaccharide‐induced inflammatory response.

## 5 CONCLUSION

In summary, MP more effectively improved the gut barrier function, regulated host immunity, and modulated the gut microbial composition compared with the single component proteins. These findings provide a theoretical basis for the selection of raw materials for clinical enteral nutrition preparations and potential use for PP waste.

## CONFLICT OF INTEREST

The authors declare no conflict of interest.
